# Neuropsychiatric Disorders in Parkinson’s Disease: What Do We Know About the Role of Dopaminergic and Non-dopaminergic Systems?

**DOI:** 10.3389/fnins.2020.00025

**Published:** 2020-01-29

**Authors:** Kathy Dujardin, Véronique Sgambato

**Affiliations:** ^1^Inserm U1171 Degenerative and Vascular Cognitive Disorders, Lille University Medical Center, Lille, France; ^2^CNRS, Institut des Sciences Cognitives Marc Jeannerod, UMR 5229, Lyon University, Bron, France

**Keywords:** depression, anxiety, apathy, psychosis, impulse control disorders, Parkinson’s disease, basal ganglia, animal models

## Abstract

Besides the hallmark motor symptoms (rest tremor, hypokinesia, rigidity, and postural instability), patients with Parkinson’s disease (PD) have non-motor symptoms, namely neuropsychiatric disorders. They are frequent and may influence the other symptoms of the disease. They have also a negative impact on the quality of life of patients and their caregivers. In this article, we will describe the clinical manifestations of the main PD-related behavioral disorders (depression, anxiety disorders, apathy, psychosis, and impulse control disorders). We will also provide an overview of the clinical and preclinical literature regarding the underlying mechanisms with a focus on the role of the dopaminergic and non-dopaminergic systems.

## Introduction

Parkinson’s disease (PD) is a neurodegenerative disease resulting from progressive death of the dopaminergic neurons in the brainstem, particularly the substantia nigra pars compacta (SNc). Besides the hallmark motor symptoms (hypokinesia, rest tremor, rigidity, and postural instability), PD patients have non-motor symptoms including neuropsychiatric disorders (Chaudhuri et al., 2006). These behavioral disorders can be separated into two categories according to their pathogenesis. Depression, anxiety, apathy, and partly psychosis are consequences of the neurodegenerative process while some psychotic symptoms and impulse control disorders (ICDs) occur as adverse effects of some antiparkinsonian drugs. In this article, we will describe the clinical manifestations of these behavioral disorders and provide some elements of epidemiology. We will also provide an overview of the current literature regarding the underlying mechanisms with a focus on the role of the dopaminergic and non-dopaminergic systems at both the clinical and preclinical levels.

## Search Strategy

A structured search in PubMed for articles written in English up to September 2019 was performed. The following keywords and MeSH terms were used: Parkinson’s disease, depression, depressive, anxiety, anxious, apathy, apathetic, psychosis, hallucination, hallucinatory, delusion, ICDs, impulsivity, impulsive, compulsion, compulsive, gambling, hypersexuality, animal model, 6-OHDA, MPTP, alpha-synuclein, pathophysiology, pathophysiological mechanisms, neurotransmission, neuromodulation, catecholamine, dopamine, dopaminergic system, dopaminergic pathway, non-dopaminergic, non-dopaminergic pathway, serotonin, norepinephrine, noradrenaline, acetylcholine, glutamate, GABA.

## Depression

The prevalence of clinically relevant depressive symptoms in patients with PD ranges from 30 to 35% according to most studies (Reijnders et al., 2008; Aarsland et al., 2011). A study on a very large sample of PD patients showed that this prevalence was higher in female than males, at advanced stages of the disease and in patients with dementia (Riedel et al., 2010).

It is noteworthy that 10 to 27% of early, untreated PD patients have significant depressive symptoms (Ravina et al., 2007b; Dujardin et al., 2014; Weintraub et al., 2015b). Moreover, depression may be a prodromal symptom of PD (Schapira et al., 2017) even though it is not a risk factor for the disease (Ascherio and Schwarzschild, 2016). In PD, there is an increased risk of depression among women, in the advanced stages of the disease and in patients with cognitive impairment (Aarsland et al., 2011).

Core symptoms of depression in PD are sadness, depressed mood, loss of pleasure, feelings of worthless, and guilt. Diagnosis is difficult because some symptoms of depression like slowness, loss of weight, sleep disturbances, or poor emotional expression are also common symptoms of PD and frequently occur in non-depressed PD patients (Okun and Watts, 2002; Brown et al., 2011). According to the last version of the Diagnostic and Statistical Manual of Mental Disorders (DSM-5), patients with PD may present minor, major or persistent depressive disorders. Depression is often comorbid with anxiety in PD (Brown et al., 2011).

The pathophysiology of depression in PD remains largely unknown and multiple factors (genetic, inflammatory, cellular regulation, signaling pathways, …) are probably incriminated. The loss of dopamine (DA) in the mesocortical and mesolimbic pathways, leading to dysfunction of the orbito-frontal area, is likely involved (Vriend et al., 2014a), as well as the loss of striatal DA, particularly in the caudate (Vriend et al., 2014b). Molecular neuroimaging studies have shown that noradrenaline (NA) (Remy et al., 2005) and serotonin (5-HT) (Boileau et al., 2008; Politis et al., 2010; Ballanger et al., 2012; Maillet et al., 2016) also play a role. The severity of depression in early untreated PD patients correlates with the reduction of the serotonergic innervation in the anterior cingulate cortex (Maillet et al., 2016). Cholinergic deficits may also be involved in PD patients with depression and cognitive deficits (Bohnen et al., 2007). Looking for blood-based biomarkers in PD, one study reported that lower plasma levels of 5-HT and its metabolite 5HIAA correlate with more severe depression in PD patients (Tong et al., 2015). However, data are few and sometimes inconsistent (Aarsland et al., 2011). Further studies are needed to determine how DA and non-DA systems interact in the regulation of mood in PD.

Most of PD patients with depression are treated with selective serotonin reuptake inhibitors (SSRIs) (Starkstein and Brockman, 2017). The efficacy of dopamine agonists to counteract depression is still controversial as pramipexole induced positive results (Barone et al., 2010) but not rotigotine (Chung et al., 2016). One clinical trial has been completed on the safety and efficacy of pimavanserin, a selective 5-HT_2A_ antagonist/inverse agonist, to treat depression in PD but there are no results available (ClinicalTrials.gov Identifier: NCT03482882). Finally, there was a clinical trial on the use of atomoxetine, a potent NA reuptake inhibitor (ClinicalTrials.gov Identifier: NCT00304161) which has been completed and gave negative results as already shown by the same group previously (Weintraub et al., 2010a).

These clinical data are to be compared with experimental data obtained in rodents (rat and mouse) and non-human primate (NHP) models of PD (see Table 1). These models afford substantial resources to investigate the etiology and pathophysiology of PD as well as to develop therapeutic approaches. Animal models of PD include acute toxin models, such as 6-hydroxydopamine (6-OHDA) or 1-methyl-4phenyl-1,2,3,6-tertrahydropyridine (MPTP), as well as genetic models, such as α-synuclein, Parkin and monoamine-related alterations. Depressive-like behavior can be observed in animals using several behavioral paradigms, such as the tail suspension test (Cryan et al., 2005) and the forced swim test (Porsolt et al., 1977). These tests have been used in animal models of PD and most of the studies showed that partial and bilateral DA lesion of the nigrostriatal pathway induced depression-like behaviors, which were predominantly counteracted by dopaminergic medication, although some studies found positive effects of SSRIs. Results are far less strong for genetic studies, which showed either no difference or an increase of depression.

**TABLE 1:** Summary of the preclinical studies investigating the pathophysiology of PD-related neuropsychiatric-like disorders.

**Behavior**	**Animal model**	**Target region (+ treatment if any)**	**Reduced/reversed by**	**Study**
Depression-like	6-OHDA-lesioned mice	Bilateral dorsolateral striatum	Pramipexole, Reboxetine	Bonito-Oliva et al., 2014
		Bilateral SNc	L-DOPA, pramipexole	Chiu et al., 2015
	6-OHDA-lesioned rats	Bilateral dorsal striatum		Branchi et al., 2008
		Bilateral medial VTA		Drui et al., 2014
		Bilateral MFB		Faggiani et al., 2015
		Bilateral MFB and 5-HT/NA lesions		Faggiani et al., 2015
		Bilateral SNc	L-DOPA, Ropinirole, SKF38393, Sumanirole, PD-128907	Carnicella et al., 2014; Drui et al., 2014
		Bilateral SNc	Ketamine, Imipramine	Vecchia et al., 2018
		Bilateral SNc		Santiago et al., 2010
		Bilateral SNc and olfactory bulb		Ilkiw et al., 2019
		Bilateral ventrolateral dorsal striatum		Tadaiesky et al., 2008; da Silva et al., 2016
		Olfactory bulb		Ilkiw et al., 2019
		Unilateral MFB		Eskow Jaunarajs et al., 2010
		Unilateral MFB	Sarizotan	Zhang et al., 2011
		Unilateral MFB	5-HT_1A_, 5-HT_6_ agonist; 5-HT_7_ agonist or antagonist	Hui et al., 2014; Liu et al., 2015; Zhang et al., 2015
		Unilateral SNc	Muscimol	Wang T. et al., 2017
		Unilateral SNc	5-HT_7_ antagonist	Han et al., 2015, 2016
		Unilateral SNc and VTA	Citalopram, L-DOPA	Winter et al., 2007
	MPTP-intoxicated mice			Vuckovic et al., 2008
			L-DOPA and D2 agonist	Mori et al., 2005
	MPTP-intoxicated rats	Bilateral SNc		Santiago et al., 2010
	D3 dopamine receptor knockout mice			Moraga-Amaro et al., 2014
	Engrailed1 heterozygote mice			Le Pen et al., 2008
	Human α-synuclein overexpressing rats	Bilateral SNc		Caudal et al., 2015
	MitoPark mice			Cong et al., 2016
	VMAT2 low expression mice			Taylor et al., 2009
Anxiety-like	6-OHDA-lesioned mice	Bilateral dorsolateral striatum	Pramipexole, Reboxetine	Bonito-Oliva et al., 2014
	6-OHDA-lesioned rats	Bilateral MFB	L-DOPA	Faggiani et al., 2018
		Bilateral SNc	SKF-38193, Sumanirole, PD-1289907	Carnicella et al., 2014; Drui et al., 2014
		Bilateral SNc		Campos et al., 2013
		Bilateral SNc	L-DOPA	Loiodice et al., 2019
		Bilateral striatum	MPEP (mGluR5 antagonist)	Chen et al., 2011a
		Bilateral striatum		Tadaiesky et al., 2008
		Unilateral MFB		Eskow Jaunarajs et al., 2010
		Unilateral MFB	L-DOPA	Zhang et al., 2011; O’Connor et al., 2016
		Unilateral MFB	5-HT_1A_ agonist, 5-HT_6_ agonist or antagonist	Sun et al., 2015
	MPTP-intoxicated mice			Gorton et al., 2010
	MPTP-intoxicated rats	Bilateral SNc		Wang et al., 2009; Sy et al., 2010
	Parkin exon 3 knockout mice			Zhu et al., 2007
	VMAT2 low expression mice			Taylor et al., 2009
	A53T α-synuclein transgenic rhesus macaques			Niu et al., 2015
Apathy-like	6-OHDA-lesioned rats	Bilateral SNc	Ropinirole, Pramipexole, PD-128907	Carnicella et al., 2014; Drui et al., 2014; Favier et al., 2014
	MPTP-intoxicated macaques			Brown et al., 2012; Tian et al., 2015
	MPTP-intoxicated macaques	(+ Bicuculline)		Sgambato-Faure et al., 2016
	VMAT2 low expression mice			Baumann et al., 2016
Psychosis-like	6-OHDA-lesioned rats	Bilateral SNc	Pimavanserin	McFarland et al., 2011; Hubbard et al., 2013
	MPTP-intoxicated macaques	(+ L-DOPA)	MDMA	Beaudoin-Gobert et al., 2015
	MPTP-intoxicated marmosets	(+ L-DOPA, Apomorphine, Pergolide, Ropinirole, Pramipexole)	Haloperidol, Clozapine, Quietapine	Fox et al., 2006; Visanji et al., 2006
Impulsive-like	6-OHDA-lesioned rats	Bilateral striatum		Tedford et al., 2015
	6-OHDA-lesioned rats	Bilateral striatum (+ Pramipexole)	Mirtazapine	Holtz et al., 2016
	Human A53T α-synuclein overexpressing rats	Bilateral SNc		Engeln et al., 2016; Jiménez-Urbieta et al., 2019
	Human A53T α-synuclein overexpressing rats	Bilateral SNc (+ Pramipexole)		Engeln et al., 2016; Jiménez-Urbieta et al., 2019
Compulsive-like	6-OHDA-lesioned rats	Bilateral SNc and VTA (+ Pramipexole)		Dardou et al., 2017
	MPTP-intoxicated macaques	(+ Bicuculline)		Sgambato-Faure et al., 2016
	MPTP-intoxicated African green monkeys			

By looking more specifically at each of these models, MPTP-treated mice (with a moderate non-selective DA lesion) exhibited an increased depressive-like behavior (Mori et al., 2005; Vuckovic et al., 2008), which was counteracted by L-DOPA or a D2 agonist (Mori et al., 2005). However, these MPTP mice studies did not specifically target the nigrostriatal or mesostriatal dopaminergic pathways and 5-HT levels were also downregulated by MPTP.

The unilateral lesion of the substantia nigra pars compacta (SNc) with the toxin 6-OHDA also induced a depressive-like behavior and a hyperactivity in the lateral habenula. Interestingly, injection of muscimol (a GABA_A_ agonist) in this area had antidepressant-like effects by decreasing the firing rate of the lateral habenula neurons and increasing serotonin release within the medial prefrontal cortex (Wang T. et al., 2017). Degeneration of the nigrostriatal pathway may impair GABA transmission in the lateral habenula, which play a role in regulating PD mood. Furthermore, the modulation of different 5-HT receptors within the lateral habenula also impacted depressive-like behavior in hemiparkinsonian rats (Han et al., 2015, 2016) and lesions of the lateral habenula cause a decrease in depressive-like behavior and an increase of 5-HT levels in the raphe nuclei (Luo et al., 2015). In association to the substantia nigra and raphe, the lateral habenula then probably contributes to the pathophysiological mechanisms of depression. After a moderate bilateral DA lesion of the SNc (noradrenergic neurons were protected from 6-OHDA with desipramine), rats had no locomotion impairment but exhibited more depressive-like behaviors which were reversed by L-DOPA, ropinirole (a D2/D3 agonist), SKF-38393 (a selective D1 agonist), sumanirole (a selective D2 agonist) and PD-128907 (a preferred D3 agonist) but not citalopram (a SSRI) (Carnicella et al., 2014; Drui et al., 2014). This suggests that the injury of the DA nigrostriatal pathway is involved in the pathogenesis of depressive-like behavior in this PD animal model. In agreement with these data, both L-DOPA and pramipexole had anti-depressant effects in the forced swim test and both normalized decreased neurogenesis in the hippocampus in mice with bilateral 6-OHDA intra-nigral lesions (Chiu et al., 2015). Administration of ketamine (an antagonist of NMDA glutamatergic receptors) or imipramine (noradrenalin and serotonin reuptake inhibitor) can also improve depressive-like behavior in rats (Vecchia et al., 2018). Another study has shown that after bilateral intranigral injection of either MPTP or 6-OHDA (leading both to a 50% DA cell loss in the SNc), rats exhibited depressive-like behaviors which were associated to hippocampal reductions of DA, 5-HT and NA, suggesting an involvement of these monoamines in depression after nigral lesions (Santiago et al., 2010). Interestingly, after a partial (around 50%) 6-OHDA lesion of DA neurons from the olfactory bulb, rats exhibited an olfactory impairment, but also depressive-like behaviors (Ilkiw et al., 2019). Moreover, this lesion exacerbated the depressive-like behaviors, classically induced by the bilateral and partial lesion of SNc (Ilkiw et al., 2019), evidencing that the degeneration of the DA pathways within the olfactory bubble also impacts depression in PD.

Regarding the potential involvement of the ventral tegmental area (VTA), conflicting results have been obtained since rats with a bilateral but partial (around 50%) lesion of the medial part of VTA displayed no motor deficits, nor depressive-like behavior (Drui et al., 2014) but anhedonia, a major symptom of depression (Dardou et al., 2014). Another study showed that combined lesions of SNc and VTA led to no motor deficits but a depressive-like behavior, whose severity increased with nigral or the VTA lesions. It was reversed by both citalopram and L-DOPA (Winter et al., 2007). This suggests that SSRIs might be efficient at least partly by modulating the DA system (Ainsworth et al., 1998; Gambarana et al., 1999).

Unilateral 6-OHDA injections into the medial forebrain bundle (MFB) can also be used to model PD in rodents even though it leads to an extensive and not selective DA lesion. Several studies have reported depressive-like behaviors in such lesioned rats without any motor deficit (Winter et al., 2007; Zhang et al., 2011). L-DOPA failed to reverse these behaviors (Eskow Jaunarajs et al., 2010) while acute administration of sarizotan, an agonist at 5-HT receptors and partial agonist at D2 DA receptors had an antidepressant effect (Zhang et al., 2011). Again, these results fit well with the known involvement of the serotonin system in the modulation of depression. Activation of prelimbic 5-HT_1__A_ receptors produced antidepressant effects while their blockade favored depressive-like behavior in the MFB-lesioned rats (Hui et al., 2014). It has also been shown that the modulation of 5-HT_6_ or 5-HT_7_ receptors within either the prelimbic cortex or the hippocampus had an impact on depressive-like behaviors (Liu et al., 2015; Zhang et al., 2015, 2016). After bilateral injection of 6-OHDA in the MFB, rats exhibited depressive-like behavior, which was dramatically exacerbated when lesions of serotonergic and noradrenergic neurons were combined with the DA one (Faggiani et al., 2015). These data emphasize again the involvement of not only DA but also other monoamine pathways in the pathophysiology of depressive-like behavior in animal models of PD.

Intra-striatal 6-OHDA injections can also be used to model PD, especially at early stages. After bilateral lesion (around 70%) of the dorsolateral striatum (with or without using desipramine to protect noradrenergic neurons), mice displayed slight motor deficits and longer immobility times in forced swim test and tail suspension test (Bonito-Oliva et al., 2014). This depressive-like behavior could be reversed by pramipexole (D2/D3 agonist) as well as by a noradrenaline inhibitor but not by L-DOPA. In another study, rats injected with bilateral 6-OHDA into the dorsal striatum (no use of desipramine), exhibited a mild reduction (36%) of striatal DA associated with increased depressive-like behaviors (Branchi et al., 2008). Other studies have shown that the partial and restricted lesion (50%) of the ventrolateral area of the dorsal striatum triggered no motor deficits but increased depressive-like behavior with monoamines alterations (Tadaiesky et al., 2008; da Silva et al., 2016). Interestingly, while anhedonia-like behaviors were observed shortly after a partial bilateral 6-OHDA lesion of the dorsolateral striatum, rats exhibited depressive-like behavior later, when anhedonia was no more present, indicating a temporal dissociation between the dorsolateral striatum and the prefrontal cortex (Matheus et al., 2016). The dorsolateral part of the striatum has motor and non-motor functions and undergoes extensive DA depletion. This leads to dysfunction of other regions such as the prefrontal cortex which has been associated with the occurrence of anhedonia and depression at premotor stages of PD.

A few studies using genetic models of PD have reported depressive-like behaviors. Mice deficient for the DA D3 receptor do not show significant deficits in locomotion when tested in the open field but exhibit depressive-like behaviors (Moraga-Amaro et al., 2014). In the same line, heterozygote mice for Engrailed 1, a developmental gene controlling the survival of DA neurons, display a progressive loss of the mesencephalic DA neurons, motor deficits as well as depressive-like behaviors (Le Pen et al., 2008). Mice deficient for the vesicular monoamine transporter-2 (VMAT-2) display enhanced depression-like behaviors, which worsen with advancing age (Taylor et al., 2009). The MitoPark mouse is a genetic model of PD, replicating several essential features of PD, including adult onset of DA neuron loss, slow progressive neurodegeneration, formation of intra-neuronal inclusions albeit without α-synuclein, responsiveness to L-DOPA treatment and non-motor deficits. MitoPark mice show a depressive-like phenotype (Cong et al., 2016). This depressive-like phenotype is observed besides several other motor and behavioral deficits and brain structural changes. Several groups have overexpressed α-synuclein in animals as a progressive model of PD (Ulusoy et al., 2010). After bilateral injection of the wild type human α-synuclein in the substantia nigra, which produces a partial DA cell loss (43% in the SNc, 30% in the VTA, 31% in the striatum), rats display depressive-like behaviors, independently of mild locomotor deficits (Caudal et al., 2015).

Up to now, no study has measured or observed a depressive-like behavior in a NHP model of PD. Such studies would be interesting since ethological investigations have shown that some monkeys are more prone to depression than others (Camus et al., 2014).

## Anxiety

**FIGURE 1:**
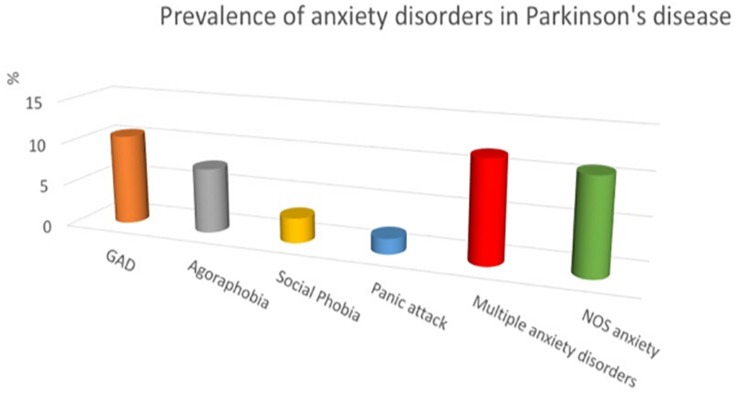
Prevalence of anxiety disorders in Parkinson’s disease (adapted from Leentjens et al., 2011). GAD, generalized anxiety disorder; NOS, not otherwise specified.

The prevalence of anxiety disorders in PD ranges from 25 to 43% (Dissanayaka et al., 2014). It is higher than in other diseases causing similar disability. A recent systematic review considering data from a total of 2399 patients found that the point prevalence of anxiety disorders in PD is 31% (Broen et al., 2016). When referring to the DSM-IV-R diagnostic criteria, generalized anxiety is the most frequent, followed by agoraphobia, social phobia and panic disorder (see Figure 1). A large proportion of patients have multiple anxiety disorders (Leentjens et al., 2011). Moreover, a recurrent observation of the studies is the large proportion of patients (13.3% according to the systematic review) with significant anxiety symptoms that do not fit usual criteria for anxiety disorders (Dissanayaka et al., 2014; Broen et al., 2016). It is considered as “not otherwise specified” (NOS) anxiety disorder (Pontone et al., 2009). It refers to PD-specific anxiety, like phobia of falling, of driving, social phobia related to potentially embarrassing symptoms (drooling, dysarthria, …), anxiety related to withdrawal of DA medication or to wearing-off of medication in patients with fluctuations, panic-like disorder related to OFF periods, among others. Adverse effects of DA medication can also generate significant anxiety as in patients with ICD, DA dysregulation syndrome, or hallucinations. By consequence, anxiety disorders are probably largely underestimated in PD as triggers (predisposing and precipitating factors) are multiple.

The main manifestations of anxiety in PD are inability to relax, feeling tense, excessive concern, restlessness. Somatic symptoms may also be observed as palpitations, shortness of breath, sweating, digestive upset, etc. Usually, all the parkinsonian symptoms worsen with anxiety. Moreover, anxiety frequently implies emotion regulation deficits, irritability, excessive tiredness and difficulties falling asleep.

The association between anxiety and motor fluctuations, the significant exacerbation of anxiety provoked by DA withdrawal suggest that neurodegeneration of the DA pathways is involved in anxiety in PD. This is also supported by a study showing that in *de novo* patients, lower DA uptake at the caudate was associated with more severe trait anxiety (Picillo et al., 2017). However, the pathophysiology of PD-related anxiety disorders is complex and remains to be elucidated. In humans, several neuroimaging studies tried to decipher the mechanisms underlying anxiety in PD. Few specifically focused on anxiety. Most were PET or SPECT studies (Remy et al., 2005; Weintraub et al., 2005; Moriyama et al., 2011; Erro et al., 2012; Ceravolo et al., 2013; Huang et al., 2013; Maillet et al., 2016; Picillo et al., 2017; Wang X. et al., 2017; Joling et al., 2018). Three were volumetric studies (Tinaz et al., 2011; Vriend et al., 2016; Wee et al., 2016). Overall, the functional studies showed the involvement of the striatum and of the DA, 5-HT and NA pathways in the occurrence of anxiety manifestations in PD. The anatomical studies showed reductions in the volume of several brain areas, namely the amygdala, the anterior cingulate cortex and the orbito-frontal cortex. However, most of these studies were correlation studies including PD patients whatever their status in terms of anxiety disorders. Moreover, the anxiety measures used were not always optimal. There is thus a real need of further investigations.

It is absolutely necessary to continue studies to decipher the mechanisms of anxiety in PD. To date, no interventional clinical trial specifically targeting PD-related anxiety has been published. Two trials have just ended but their results are not published. One concerns rotigotine, a D2–D3 agonist (ClinicalTrials.gov Identifier: NCT02365870). The other concerns buspirone, a 5-HT_A__1_ agonist (ClinicalTrials.gov Identifier: NCT02803749).

Anxiety-like behaviors can be observed in animals using several behavioral paradigms such as the elevated plus maze, the Light/Dark box and the open field. These tests have been used in animal models of PD and most of the literature report an increased anxiety-like behavior in 6-OHDA- or MPTP-lesioned animals (Table 1). In contrast, genetic models of the disease either show no difference or a decrease of anxiety-like behavior.

Specifically, after a MPTP intoxication that causes an almost complete depletion of striatal DA, mice exhibited motor deficits and an increased anxiety-like behavior, concomitant with a reduction of 5-HT levels in the basolateral nucleus of the amygdala (BLA) (Gorton et al., 2010). Several studies also reported an increase in anxiety-like behavior after bilateral intranigral injection of MPTP in rats (Wang et al., 2009; Sy et al., 2010).

After 6-OHDA bilateral lesion of nigral neurons within the SNc (inducing a maximal DA loss of about 70% in the dorsal striatum, noradrenergic neurons being protected with desipramine), rats exhibited no motor deficits but an increased anxiety-like behavior (Carnicella et al., 2014; Drui et al., 2014). A significant correlation was found between the latency of response and the striatal DA loss, suggesting that the increased anxiety-like response was related to the degree of striatal DA depletion (Drui et al., 2014). However, no similar correlations were found in the elevated plus-maze. DA agonists (SKF-38193, Sumanirole, and PD-128907) all reduced anxiety-like behavior in these 6-OHDA bilaterally lesioned rats (Carnicella et al., 2014). Another study showed that after a partial (less than 45%) and bilateral 6-OHDA lesion of the SNc (desipramine use), rats displayed an increased anxiety-like behavior as well as motor deficits (Campos et al., 2013). Bilateral partial (48%) lesion of the SNc induces significant deficits in the elevated plus maze, which were not reversed by either acute or chronic treatment with L-DOPA (Loiodice et al., 2019). This lesion was not associated to motor impairment.

The partial and bilateral lesion of the medial VTA failed to induce anxiety (Drui et al., 2014). On the contrary, after unilateral 6-OHDA MFB lesion, rats exhibited a mild increase of anxiety-like behavior, which could not be improved by chronic L-DOPA (Eskow Jaunarajs et al., 2010). However, other studies using the same type of lesion have shown anxiety-like behaviors, which could be improved by chronic L-DOPA or diazeapam (O’Connor et al., 2016), but not by sarizotan (Zhang et al., 2011) or diazepam (O’Connor et al., 2016). Several studies have shown that acting on 5-HT_1__A_, 5-HT_6_ or 5-HT_7_ receptor subtypes within the amygdala, the hippocampus or the prelimbic cortex can modulate anxiety-like behavior (Sun et al., 2015, 2018; Du et al., 2018; Zhang et al., 2018; Liu et al., 2019), underlying the major role of the serotonergic system in anxiety-like behavior in PD animal models. However, another study has shown, in 6-OHDA bilaterally MFB lesioned rats, that the additional depletion of serotonin or of noradrenalin had no further effects on anxiety-like behavior (Faggiani et al., 2015). However, L-DOPA enhanced the firing rate of amygdala and significantly decreased anxiety in these animals (Faggiani et al., 2018). This suggests that the increased activity of serotonin neurons may enhance the anxiolytic action of L-DOPA.

In mice, bilateral 6-OHDA lesion (75%) of the dorsolateral striatum also led to an increased anxiety-like behavior with slight motor deficits (Bonito-Oliva et al., 2014). This anxiety-like behavior was corrected by pramipexole (but not by L-DOPA) and reboxetine (a selective noradrenaline reuptake inhibitor), and was independent of noradrenaline depletion as the use of desipramine to protect noradrenergic neurons from the toxicity of 6-OHDA did not modify this anxiety-like behavior (Bonito-Oliva et al., 2014). Again, this suggests that the depletion of DA caused by 6-OHDA is sufficient to induce affective-like symptoms. Other studies showed increased anxiety in rats with a bilateral and partial (less than 50%) lesion of the striatum inducing slight motor deficits as well (Tadaiesky et al., 2008; Chen et al., 2011a; da Silva et al., 2016). The administration of an antagonist of metabotropic receptors was efficient to counteract both behavioral and neuronal changes (Tadaiesky et al., 2008; Chen et al., 2011a, b; da Silva et al., 2016).

A few studies have reported anxiety-like symptoms in genetic mouse models of PD. For instance, Zhu and colleagues reported increased anxiety-like behavior in Parkin deficient mice (Zhu et al., 2007). VMAT-2 deficient mice display an enhanced anxiety-like behavior, worsening with aging (Taylor et al., 2009). Compared to α-synuclein deficient mice and wild-type controls, mice overexpressing the human mutated form A53T of α-synuclein exhibited, besides early and late stage cognitive and sensorimotor deficits, a reduced anxiety-like behavior (George et al., 2008). These results indicate a possible role for α-synuclein in anxiety-like behavior.

Regarding studies performed on monkey models of PD, only one study mentioned the case of a transgenic macaque monkey expressing the mutated form of α-synuclein and exhibiting an enhanced anxiety-like behavior (Niu et al., 2015).

## Apathy

Clinically, apathy refers to a set of behavioral, emotional and cognitive manifestations, such as reduced interest and participation in the main activities of daily life, loss of initiative, lack of perseverance, indifference, and flattening of affect. As noted by Marin, apathy can exist *per se* as a syndrome. It is not just a symptom of depression or dementia (Marin, 1991). The definition of Marin was anchored on the motivational component of apathy: “*diminished motivation not attributable to diminished level of consciousness, cognitive impairment, or emotional distress*” (Marin, 1996). Due to the difficulty of addressing the concept of motivation, a more operational definition of apathy is currently preferred. According to the recent revision of the clinical diagnosis criteria (see Table 2), apathy corresponds to a quantitative reduction of goal-directed activity either in behavioral, cognitive, emotional, or social dimensions in comparison to the patient’s previous level of functioning in these areas (Robert et al., 2018).

**TABLE 2:** Criteria for clinical diagnosis of apathy.

**Criterion A:** A quantitative reduction of goal-directed activity either in behavioral, cognitive, emotional, or social dimensions in comparison to the patient’s previous level of functioning in these areas. These changes may be reported by the patient themselves or by observation of others.
**Criterion B:** The presence of at least 2 of the 3 following dimensions for a period of at least 4 weeks and present most of the time.
B1. BEHAVIOR AND COGNITION
**Loss of, or diminished, goal-directed behavior or cognitive activity** as evidenced by at least one of the following: – reduced level of activity either at home or work, makes less effort or needs to be prompted to perform activities; – less persistence in maintaining an activity or conversation, finding solutions to problems or alternative ways; – less interest in or reaction to news, or less interest in doing new things; – less interest in their own health and well-being or personal image;
B2. EMOTION
**Loss of, or diminished, emotion** as evidenced by at least one of the following: – less spontaneous (self-generated) emotions regarding their own affairs; – less emotional reaction in response to positive or negative events in the environment, – less concern about the impact of their actions or feelings on the people around him/her; – less empathy to the emotions or feelings of others; – less verbal or physical reactions that reveal his/her emotional states;
B3. SOCIAL INTERACTION
**Loss of, or diminished engagement in social interaction** as evidenced by at least one of the following: – less initiative in spontaneously proposing social or leisure activities to family or others; – less participation in social or leisure activities suggested by people around them; – less interest in family members; – less likely to initiate a conversation, or early withdrawal from it; – less interest in getting out to meet people;
**Criterion C:** These symptoms (A–B) cause clinically significant impairment in personal, social, occupational, or other important areas of functioning.
**Criterion D:** The symptoms (A–B) are not exclusively explained or due to physical disabilities (e.g., blindness and loss of hearing), to motor disabilities, to a diminished level of consciousness, to the direct physiological effects of a substance (e.g., drug of abuse, medication), or to major changes in the patient’s environment.

According to a meta-analysis on a large set of data, the mean prevalence of apathy in PD is 39.8% (*n* = 5,388; 95% CI 34.6–45.0%) (den Brok et al., 2015). Patients with apathy are on average older than the non-apathetic patients, have more impaired cognition in general and executive function in particular, have more severe motor symptoms and an increased risk of co-morbid depression. Apathy may be a predictive factor for dementia and cognitive decline over time (Dujardin et al., 2009; Pedersen et al., 2009).

Regarding the mechanisms underlying apathy, most neuropathological and neuroimaging studies in apathetic patients show abnormalities in a network of brain regions involved in motivated behavior, especially the dorsal part of the anterior cingulate cortex, the medial orbitofrontal cortex and the ventral striatum, under the influence of the mesolimbic DA system originating in the VTA (Heron et al., 2018). However, only few studies have examined the pathophysiology of apathy in PD patients. Reijnders et al. (2010) used voxel-based morphometry to identify the anatomical correlates of apathy in a group of 60 PD patients (16% were apathetic). High apathy scores were correlated with lower cognitive efficiency, more depressive symptoms and low gray matter density in the posterior cingulate, precuneus, insula and inferior parietal gyrus. Carriere et al. (2014) compared with shape analysis the volume of the striatum in apathetic vs. non-apathetic PD patients and showed a remodeling of the left caudate and left accumbens in apathetic patients. Most other studies in PD have used functional imaging. Remy et al. (2005) used [11C]-RTI-32 Positron emission tomography (PET), an *in vivo* marker of both DA and NA membrane transporters, with low affinity for 5-HT transporters. They found that apathy scores were negatively correlated with binding potential values in the ventral striatum bilaterally, suggesting that catecholamine denervation of this area has a role in apathy. We used [11C]DASB and [11C]PE2I PET, radioligands, binding to the serotonergic and dopaminergic transporters, respectively (Maillet et al., 2016) and found that the severity of apathy in 12 *de novo* PD patients was mainly related to specific serotonergic lesions within the right-sided anterior caudate nucleus and the orbitofrontal cortex. Robert et al. (2012) used ^18^FDG PET to measure glucose metabolism in 45 patients with PD and reported a correlation between the severity of apathy symptoms and cerebral metabolism in the inferior frontal gyrus, middle frontal gyrus, cuneus and anterior insula, at the right side. As apathy is a possible complication of the treatment of PD with deep brain stimulation of the subthalamic nucleus (STN-DBS), several studies have used the model of STN stimulation to investigate the pathophysiology of apathy in PD. Jeune et al. (2009) observed a worsening of apathetic symptoms after surgery and showed that this change was correlated with greater glucose metabolism in the right frontal middle gyrus (Brodmann area 10) and right inferior frontal gyrus (Brodmann areas 46 and 47) and lower glucose metabolism in the right posterior cingulate gyrus (Brodmann area 31) and left medial frontal lobe (Brodmann area 9). According to Jeune et al. (2009), stimulation of the STN could have a negative effect on the limbic territory of the STN, thus destabilizing the limbic circuit. The same team showed that reduced preoperative glucose metabolism within the right ventral striatum was associated with the occurrence of post-surgery apathy (Robert et al., 2014).

Thobois et al. (2010) used PET with an antagonist of DA D2 receptors to investigate the role of DA denervation in post-surgery apathy. They compared 12 patients who had developed apathy after initiation of DBS and 13 control PD patients who had not developed apathy after stimulation. Binding potential values were higher in apathetic patients compared with non-apathetic patients, in the orbito-frontal, dorsolateral and posterior cingulate cortex, bilaterally, as well as in the left striatum and right amygdala. After a challenge with methylphenidate, a drug that inhibits the reuptake of DA and NA and increases DA concentration at dopaminergic terminals, they observed that apathetic patients had lower reserve of endogenous DA. Although these results are consistent with a hypodopaminergic etiology of apathy in PD, other neurotransmitter systems are probably involved. As already mentioned, we found a prominent role of serotonergic degeneration in the occurrence of apathy at early stage of the disease (Maillet et al., 2016). This was confirmed by a complementary study by Prange et al. (2019) showing microstructural changes specifically associated with apathy in the limbic system in *de novo* patients with apathy compared with non-apathetic patients and healthy controls. These changes overlapped with the functional alteration of the serotoninergic terminals but not with the dopaminergic abnormalities (Prange et al., 2019). Given the links between apathy and cognitive decline in PD patients, a role of cholinergic denervation is also possible. The reduction of apathy symptoms by administration of rivastigmine, an inhibitor of acetylcholinesterase, reinforced such an assumption (Devos et al., 2013). Further confirmation of the role of non-dopaminergic systems in the PD apathy is still needed.

The first studies searching for a pharmacological treatment of apathy targeted the dopaminergic pathway. Czernecki et al. (2008) were the first to describe an improvement of post-surgery apathy after administration of ropinirole in a small group of patients. However, the level of evidence was very low. Later, Thobois et al. (2013) tested the effectiveness of piribedil to reduce post-surgery apathy in PD. They observed a significant reduction of the symptoms after 12 weeks of pharmacotherapy (Thobois et al., 2013). The effectiveness of rotigotine, another D2/D3 dopamine agonist, on apathy was also tested but there was no effect on the symptom severity (Hauser et al., 2016). Methylphenidate, a drug enhancing mesolimbic dopaminergic stimulation, was shown to improve apathy in an 82-year-old patient. Its effectiveness was not explored further in PD (Chatterjee and Fahn, 2002). As stated above, the efficacy of rivastigmine, an acetylcholinesterase inhibitor, on apathy in non-demented, non-depressed patients was also tested (Devos et al., 2013). Compared with placebo, there was a significant improvement of apathy in the rivastigmine group, particularly on the cognitive and initiation components of apathy.

Apathy is difficult to assess in animals. Some behavioral conditions considered as “activities of daily life” can be used in rodents such as the burrowing test or the nest building test. Other tests can also be used such as the runway task for food, operant sucrose self-administration and the saccharin preference test. There are very few studies on apathy-like behavior in PD animal models (Table 1). Three studies from the same group have evidenced motivational deficits after the selective, partial (less than 70%) and bilateral 6-OHDA lesion of the SNc in rats (Carnicella et al., 2014; Drui et al., 2014; Favier et al., 2014). But these animals also exhibited depressive-like and anxious-like behaviors. This apathy-like behavior, which was detected with the operant sucrose self-administration, was only improved by drugs preferentially acting on D3 type of DA receptors such as ropinirole, pramipexole, and PD-128907. Lesion of the medial part of VTA did not induce such a behavior, indicating that damage of the nigrostriatal pathway is a key risk factor to develop apathy-like behavior in the 6-OHDA rat model of PD. Another study did report behavioral signs of apathy in aged mice deficient for the vesicular monoamine transporter 2 (Baumann et al., 2016).

We have shown that apathy-like behavior (a hypoactive state with loss of motivation for food) can be induced by non-DA pharmacological dysfunction of the primate ventral striatum (Sgambato-Faure et al., 2016; Voon et al., 2017; Sgambato and Tremblay, 2018). This behavior involved a circuit including the orbital and medial prefrontal cortex, anterior insula, and lateral parts of medial output basal ganglia regions. This lack of motivation was induced by bicuculline (a GABA_A_ antagonist) in moderately DA-depleted monkeys, suggesting that dopamine might only modulate the expression of apathy rather than cause it. However, another group has shown in MPTP-treated monkeys that the dopaminergic pathways play a key role in apathy-like behavior (Brown et al., 2012). Specifically, they tested the impact of MPTP on the monkeys’ willingness to attempt goal-directed behaviors (Brown et al., 2012). Using PET imaging and post-mortem analysis, they showed that apathy-like scores correlated with the degree of lesion of the DA mesostriatal pathway, and that dysfunction of the mesostriatal pathway predicted apathy-like behavior better than DA nigrostriatal dysfunction. More recently, the same group has extended these findings by showing that DA dysfunction in cortical regions also contributed to the development of apathetic behavior in NHP (Tian et al., 2015). Apathy scores correlated with DA injury (detected by PET tracers) in the dorsolateral prefrontal cortex, ventromedial prefrontal cortex, and insular cortex. Furthermore, only VTA cell counts could predict DAT changes in the insular cortex, suggesting a particular role for this pathway in the manifestation of apathy in this monkey model of PD. Contrary to the results in rodents, these data indicate a key role for the VTA involving both subcortical and cortical regions in the pathophysiology of apathy. Further studies investigating the role of both DA and non-DA pathways in apathy-like behavior in PD animal models are thus warranted.

## Psychosis

In PD, psychosis encompasses a set of symptoms including illusions, hallucinations, delusions, and related symptoms. These symptoms are typically visual, more rarely in other modalities. They usually form a continuum progressing with the course of the disease (Ffytche and Aarsland, 2017). Passage (feeling like something is passing at the outer visual field) and presence (feeling that someone is close by) hallucinations as well as illusions (mis- or distorted perception of an actual stimulus) are the most common symptoms at early stage of the disease. Passage and presence hallucinations may concern 50% of the patients (Wood et al., 2015). As the disease progresses, visual hallucinations in PD may be complex and formed, usually vision of animals, people or objects. The scene is often stereotyped. They tend to occur in a low sensory environment. The hallucinations usually ignore the patient and disappear when approaching. Initially, insight is preserved but later, it is lost and contribute to the development of delusions.

There are few prevalence studies and frequencies vary widely. Point prevalence of complex hallucinations ranges from 22 to 38% (Fenelon and Alves, 2010). The prevalence of minor psychotic symptoms is much more variable. Fénelon et al. (2000) reported that 26% of a sample of 216 consecutive patients taking medication had minor hallucinations. In early diagnosed untreated patients, we observed that 6.32% had minor hallucinations (Dujardin et al., 2014) while another study reported a much higher prevalence of 42% (Pagonabarraga et al., 2016). The risk of developing visual hallucinations increases with age, severity of motor and cognitive symptoms, disease duration and medication (Fenelon and Alves, 2010). Moreover, visual hallucinations have been shown to be the main risk factor for nursing home placement (Aarsland et al., 2000). It increases the risk of cognitive impairment and dementia (Guo et al., 2019) and mortality (Goetz and Stebbins, 1995).

The mechanisms behind visual hallucinations (VH) remain largely unknown. Changes in visual information processing are incriminated. For example, compared with patients without visual hallucinations and healthy controls, PD patients with visual hallucinations had a thinning of the retinal nerve fiber layer, as measured by optical coherence tomography (Lee et al., 2014). Some studies have shown that PD patients with visual hallucinations have lower performance in visuospatial tasks, but findings are not consistent probably due to a lack of sensitivity of the tests and variability of the populations (for a review, see Ffytche and Aarsland, 2017). However, changes in the networks involved in perceptual and cognitive processing of visual information have been shown by anatomical and functional neuroimaging studies in patients with visual hallucinations compared with those without visual hallucinations. Most anatomical studies reported an atrophy in areas corresponding to dorsal and ventral visual streams (for a review, see Ffytche and Aarsland, 2017). Functional MRI (fMRI) studies reported reduced activity of the visual areas compensated by an increased activity in the associative cortical areas (Diederich et al., 2009; Shine et al., 2012; Yao et al., 2014, 2015, 2016; Shine et al., 2015). However, a better understanding of the pathophysiology of chronic visual hallucinations in PD lacks a direct exploration of the brain changes at the time of hallucination (i.e., capture studies). Because of the challenging nature of state explorations of hallucinations, only two studies were in PD. One single-case study reported the results of an fMRI scan performed in a 66-year old PD patient at time of hallucinations (Goetz et al., 2014). When comparing epochs with and without VH, there was an increased activation in a large attentional network and decreased activation in the right primary visual cortex. Recently, we measured brain functional changes when VH occurred in seven PD patients. We observed increased connectivity in visual networks concomitant to VH (Dujardin et al., 2019).

According to clinical experience, DA agonists may cause the onset of hallucinations, suggesting a role of the dopaminergic pathways in the occurrence of psychosis. However, the role of medication is very controversial since visual hallucinations are observed even in drug-naïve patients. As suggested by a work group on psychosis in PD, anti-parkinson medication is more to consider as a modifier than a trigger of hallucinations (Ravina et al., 2007a; Dujardin et al., 2014; Weintraub et al., 2015a). Regarding the other neuromodulation systems, the benefit of acetylcholinesterase inhibitors on symptoms suggests an involvement of the cortical cholinergic pathway (Burn et al., 2006; Oh et al., 2015). Moreover, an atrophy of the substantia innominate has been observed in PD patients with hallucinations (Shin et al., 2012). A contribution of 5-HT has also been suggested since a 5-HT depletion in the ventral occipitotemporal regions and bilateral frontal cortex was found in PD patients with visual hallucinations (Ballanger et al., 2010). However, further investigations are really needed to better understand the respective role of DA and non-DA pathways in the occurrence of psychosis in PD.

Pharmacological treatments currently available to reduce psychosis in PD patients have an action on these DA and 5-HT pathways. Benefits on psychotic symptoms have been reported in several placebo-controlled trials with clozapine and pimavanserin while quietapine and olanzapine provided no significant benefit (Wilby et al., 2017).

The number of studies in which psychosis-like symptoms have been observed in animal models of PD is still limited (Table 1). Behavioral assays such as head switches, hallucinatory-like responses, amphetamine-induced locomotor activity and disrupted prepulse inhibition are frequently used to assess antipsychotic activity. These tests have been used in animal models of PD.

Animals with bilateral 6-OHDA lesions of the substantia nigra display motor impairments as well as increased head twitches, locomotor activity, and disrupted prepulse inhibition (McFarland et al., 2011; Hubbard et al., 2013). Pimavanserin, a selective 5-HT_2__A_ antagonist/inverse agonist, reverse psychosis-like behaviors, suggesting that 5HT_2__A_ antagonism/inverse agonism may treat psychosis in PD.

A psychosis-like behavior rating scale has been developed and used in MPTP-treated marmosets (Fox et al., 2006; Visanji et al., 2006). This scale allows to assess four types of psychosis-like behaviors: agitation, hallucinatory-like responses to non-apparent stimuli, obsessive grooming, and stereotypies. It has been shown that L-DOPA, apomorphine, pramipexole, pergolide, ropinirole reversed motor symptoms and induced psychosis-like behaviors in MPTP-intoxicated marmosets, and that amantadine exacerbated them (Fox et al., 2006; Visanji et al., 2006). Finally, while haloperidol reduced those behaviors but increases motor symptoms, clozapine and quietapine reduced them without exacerbating parkinsonian disability. We have shown that L-DOPA triggered such psychosis-like behaviors in moderately-lesioned macaque monkeys and that these psychosis-like behaviors were reduced following lesion of serotonergic fibers by MDMA (Beaudoin-Gobert et al., 2015). Altogether, these data suggest both DA, 5-HT and glutamatergic mechanisms are involved in the pathophysiology of psychosis-like behavior.

## Impulse Control Disorders

Impulse control disorders refer to a class of psychiatric disorders characterized by impulsivity, i.e., an urge or failure to resist to temptation. In the DSM-5, it is included in a new section labeled “disruptive, impulse control, and conduct disorders.” ICDs result in behaviors performed repetitively, excessively, and compulsively to an extent that interferes with major areas of daily life (Weintraub and Claassen, 2017). The individual pursues certain reward-based activities without taking account of the potentially deleterious consequences of these repetitive activities. The semiology is diverse. In PD, four major forms of ICDs have been described: pathological gambling, compulsive buying, pathological sexual behavior, and compulsive eating. Other impulsive/compulsive behaviors are linked, namely DA dysregulation syndrome (Giovannoni et al., 2000), an addiction to DA medication, particularly high-potency and short-acting drugs (e.g., subcutaneous apomorphine or dispersible formulation of levodopa). Related phenomena have also been identified as punding (repetitive, purposeless, and stereotyped behaviors) (Spencer et al., 2011), hobbyism (excessive exercise or creative activities) and hoarding (acquisition and keeping of a large number of items with little or no objective value, e.g., used gloves) (O’Sullivan et al., 2010). Very often, those patients also develop hypomania which interferes at lot with their familial and social life.

Regarding prevalence, the main available data come from the DOMINION study, which included 3090 PD patients from movement disorders clinics in North America and assessed the frequency of the four main ICDs (Weintraub et al., 2010b). It revealed that 13.6% of patients had one or more ICDs (compulsive buying in 5.7%, gambling in 5%, binge eating disorders in 4.3% and compulsive sexual behavior in 3.5%) and 3.9% had two or more ICDs. Similar prevalence data have been reported in studies conducted in different countries around the world (for a review, see Weintraub and Claassen, 2017). The Italian multi-center prospective ICARIUS study reported a higher point prevalence with 28.6% of the 1069 patients having at least one ICD (Antonini et al., 2017). The 2-year incidence was of 20.6% (Barone et al., 2019). The prevalence of the other impulsive/compulsive disorders is not well-documented.

Dopamine replacement therapy, namely DA agonist use, is the main risk factor for ICDs. In the DOMINION study, ICDs were more frequent in patients receiving DA agonists (17.1%) than in patients only treated by levodopa (6.9%). An analysis on the baseline data of the Parkinson’s Progression Markers Initiative (PPMI) cohort, including 168 untreated PD patients and 143 healthy control subjects, revealed that the frequencies of ICDs and related symptoms did not differ in both groups (Weintraub et al., 2013). After 24-month follow-up, there was no increase of ICDs prevalence in the PD patient group, overall. However, in those who were on DA replacement therapy for at least 1 year, the incidence of ICDs was significantly higher than in patients still untreated or who initiate DA replacement therapy for less than 1 year (de la Riva et al., 2014). Hence, these results suggest that PD itself does not increase the risk to develop ICDs but reinforce the hypothesis that DA medication plays a major role in the occurrence of such disorders. The risk is greater with DA agonists having a preferential selectivity for D3 and D2 receptors, suggesting that the mesocortical and mesolimbic dopaminergic pathways are most likely involved. These pathways play a key role in reward-based learning and decision-making (Ballard et al., 2011; Haber, 2014; Hauser et al., 2017). Dysfunction of this DA system was confirmed by several neuroimaging studies (Cilia et al., 2008; Steeves et al., 2009; Voon et al., 2010, 2014). However, ICDs seem to be multi-determined and dysfunction of the mesocorticolimbic system due to DA replacement therapy is not the only underlying mechanism. Other clinical features (early onset disease, comorbid depression, and anxiety), premorbid (personality traits, genetic polymorphisms, past history of addiction), environmental (ease to access to the internet, pornography, casino, etc.), demographical (higher frequency of hypersexuality in men than women while the inverse is observed for compulsive buying) and cultural factors, cognitive bias (altered executive functions and decision-making) also influence the occurrence of ICDs (Weintraub et al., 2015b). Other neuromodulation systems are probably also involved, as suggested by studies in animal models of PD but further investigations are needed in patients.

To date, there is no available treatment for ICDs (Gatto and Aldinio, 2019). One clinical trial should recruit PD patients soon. It concerns pimavanserin, a selective 5-HT_2__A_ inverse agonist (ClinicalTrials.gov Identifier: NCT03947216). Another trial has started recruiting PD patients. It will test the efficacy of clonidine, an α2 noradrenergic agonist (ClinicalTrials.gov Identifier: NCT03552068).

The number of studies in which impulsive/compulsive-like behavior has been modeled in animal models of PD is still quite limited (Table 1). Several models try to recapitulate the DA deficit of early or more advanced PD and the impulsive or compulsive traits provoked by DA loss in association with dopatherapy, in order to better understand pathophysiological mechanisms. Impulsive or compulsive behaviors can be observed in animals using several behavioral tasks, such as food-related instrumental learning tasks (5-choice reaction time task, delay-discounting task, rat gambling task, rodent betting task, etc.). Some of these tests have been used in animal models of PD.

Specifically, the lesion of the DA pathway seems to be necessary but not always sufficient to induce impulsive/compulsive-like behaviors, in agreement with the clinical study mentioned above, showing that the DA lesion by itself does not confer any risk for ICDs in *de novo* PD patients (Weintraub et al., 2013). On the 6-OHDA rat model of PD, it has been shown that bilateral dopaminergic lesions (use of desipramine) of the dorsolateral striatum increase impulsivity using a delay-discounting task (Tedford et al., 2015). Delivery of pramipexole via osmotic pumps enhanced impulsivity (risk-taking), which is reduced by mirtazapine, a NA and 5-HT antidepressant in those moderately-lesioned rats (Holtz et al., 2016). Moreover, the administration of chronic pramipexole to bilaterally 6-OHDA-lesioned rats (75% of DA lesion in the SNc and 50% in lateral part of VTA; with desipramine pretreatment; minor motor impairment) was required to induce a compulsive-like behavior (using a lever-pressing task), the DA lesion by itself being without any effects (Dardou et al., 2017). Furthermore, mapping performed with the expression of the immediate early gene c-*fos* suggests that the behavior is supported by the activation of the orbitofrontal cortex and the dorsal striatum (Dardou et al., 2017). This is consistent with previous studies showing that activation (Ahmari et al., 2013) or inhibition (Burguière et al., 2013) of the orbitofrontal cortex–striatal pathway modulates repetitive grooming in mice. In normal unlesioned rats, chronic pramipexole failed to induce compulsive behavior (Dardou et al., 2017). By contrast, quinpirole administration does induce compulsive checking behavior and is used as a rat model of obsessive-compulsive disorders (Winter et al., 2008). Compulsive behavior may share similarities with habit formation (Graybiel and Rauch, 2000) and lesion of the striatum is known to increase perseverative behavior in rodents on a 5-choice serial reaction time task in rodents (Baunez and Robbins, 1999).

Regarding genetic models of PD, it has been shown recently that the bilateral SNc lesion (64% of loss considered as mild) in rats overexpressing human A53T mutated α-synuclein, enhanced waiting impulsivity (increases of the premature response rate using the 5-choice serial reaction time task) and that this behavior was further exacerbated with long-term administration of pramipexole in both OFF and ON states (Jiménez-Urbieta et al., 2019). By contrast, pramipexole was not associated with changes in compulsivity. Similarly, α-synuclein-induced nigrostriatal neurodegeneration increases impulsivity by itself, subsequent chronic pramipexole administration exacerbating it (Engeln et al., 2016). Impulsive behavior does not develop in 6-OHDA-lesioned rodents (Baunez and Robbins, 1999; Carvalho et al., 2017). Differences may rely on the severity and topography of the dopaminergic lesions and on the tests used.

We have shown that impulsive and compulsive behaviors can be induced by pharmacological perturbation of different basal ganglia territories in NHP (for a review, see Sgambato and Tremblay, 2018). Compulsive behaviors were exhibited by both normal and DA-moderately lesioned monkeys. They involved a circuit including the lateral orbitofrontal cortex and limbic parts of the basal ganglia (Sgambato-Faure et al., 2016). Further studies are needed to investigate pathophysiological mechanisms of such behavioral disorders in parkinsonian monkeys.

## Limitations of the Existing Animal Models of Pd-Related Neuropsychiatric Disorders and Future Directions

Most of the existing animal models on PD rely on the use of neurotoxins (Cenci and Sgambato, 2019). 6-OHDA is used in mice and rats, while MPTP is mainly used in mice and monkeys (although it can also be used in rats). These neurotoxic models have been used extensively in the past to study motor aspects related to PD. As research now also aims at studying non-motor symptoms, these models have important limitations because the DA lesion of the nigrostriatal system often induces a decrease in motor abilities. Moreover, these neurotoxic models do not reproduce all the pathological features of the disease such as the formation of protein aggregates resembling Lewy bodies, or the involvement of brain regions outside of the nigrostriatal DA system. The same models are used to study the pathophysiological mechanisms of different neuropsychiatric-like disorders, without any specificity. There is therefore a real need to evolve these animal models, with more specific tools and/or combined lesions or impairments, to better understand the dysfunctions associated to each neuropsychiatric symptom. An important advance has been made with the development of genetic models of PD, including those based on the use of alpha-synuclein. This abnormally folded protein is enriched in Lewy bodies, the pathological sign of the disease. The injection and overexpression of this protein (in its native or mutated form) in the substantia nigra induces a more progressive loss of DA neurons. This offers the possibility of a longer asymptomatic motor phase to evaluate neuropsychiatric symptoms, without interference with motor signs. This is interesting in the case of depression and anxiety, for example, which may occur during the prodromal phase of PD. Moreover, we must really diversify our models to be able to mimic different stages of progression of the disease and the associated symptoms.

Finally, we have summarized in Table 3 the current knowledge regarding the potential involvement of the neurotransmission systems in the pathophysiology of neuropsychiatric disorders through the different species used (rodent and primate). It comes out that, although the DA and 5-HT systems seem to be involved in the pathophysiology of all these symptoms, the other systems have been much less studied. For example, in PD patients, there is evidence for an involvement of the cholinergic system in depression, apathy and psychosis but, to our knowledge, no animal study has yet shown causal links between an alteration of this system and the expression (or the modulation) of a neuropsychiatric-like symptom. Furthermore, there is a real need for new preclinical studies on the pathophysiological substrates of apathy, psychosis and ICD as the arsenal of drugs to treat these disorders in patients is limited or non-existent (for ICD).

**TABLE 3:** Overview on the involvement of DA and non-DA systems in the pathophysiology of neuropsychiatric disorders through the different species used.

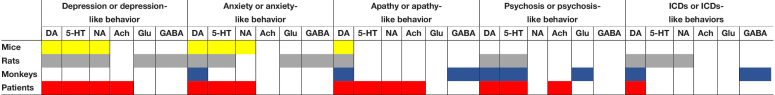

*Yellow, gray, blue, and red colors respectively refer to studies performed on mice, rats, monkeys, or patients.*

## Conclusion

Neuropsychiatric disorders are among the most common and disabling non-motor manifestations of PD. Their negative impact on quality of life of both the patients and caregivers is indubitable. They also result in a heavy socio-economic cost. In spite of that, the underlying mechanisms remain largely unknown. Available data suggest that both dopaminergic and non-dopaminergic (serotonergic, noradrenergic, cholinergic, glutamatergic, and GABAergic) systems are involved in the expression or modulation of these disorders. Translational studies with valid animal models of PD and well-characterized group of patients are needed to continue deciphering the affected processes and propose efficient therapeutic strategies.

## Author Contributions

Both authors wrote the draft and reviewed the manuscript.

## Conflict of Interest

The authors declare that the research was conducted in the absence of any commercial or financial relationships that could be construed as a potential conflict of interest.
